# Antigen Presentation by MHC-Dressed Cells

**DOI:** 10.3389/fimmu.2014.00672

**Published:** 2015-01-05

**Authors:** Masafumi Nakayama

**Affiliations:** ^1^Frontier Research Institute for Interdisciplinary Sciences, Tohoku University, Sendai, Japan

**Keywords:** intercellular communication, trogocytosis, exosomes, dressing, MHC

## Abstract

Professional antigen-presenting cells (APCs) such as conventional dendritic cells (DCs) process protein antigens to MHC-bound peptides and then present the peptide–MHC complexes to T cells. In addition to this canonical antigen presentation pathway, recent studies have revealed that DCs and non-APCs can acquire MHC class I (MHCI) and/or MHC class II (MHCII) from neighboring cells through a process of cell–cell contact-dependent membrane transfer called *trogocytosis*. These MHC-dressed cells subsequently activate or regulate T cells via the preformed antigen peptide–MHC complexes without requiring any further processing. In addition to trogocytosis, intercellular transfer of MHCI and MHCII can be mediated by secretion of membrane vesicles such as exosomes from APCs, generating MHC-dressed cells. This review focuses on the physiological role of antigen presentation by MHCI- or MHCII-dressed cells, and also discusses differences and similarities between trogocytosis and exosome-mediated transfer of MHC.

## Introduction

Intercellular transfer of MHC was first observed by Cone et al. over 40 years ago (1). In this study, mouse T cells were adoptively transferred to MHC-mismatched mice, and the authors surprisingly found the MHC of the recipient mice on transferred T cells (1). Since this seminal study, numerous others have shown that the T cell receptor (TCR) rapidly (within minutes) acquires MHC molecules from antigen-presenting cells (APCs) via the immunological synapse formed at cell–cell contact area, and that this phenomenon impacts T cell activation (2–8), although the physiological relevance of this is still not fully understood. This intercellular transfer of plasma membrane has been called absorption, acquisition, nibbling, shaving, snatching, stripping, or trogocytosis, which is from the ancient Greek *Trogo*, meaning “gnaw” (9, 10). Among these names, trogocytosis is now most commonly used. Several recent studies reported that trogocytosis of MHC class I (MHCI) and MHC class II (MHCII) occurs not only between T cells and APCs, but between a wide variety of cell types including APCs–APCs, APCs–natural killer (NK) cells, tumor cells–T or NK cells, etc. (10–14), suggesting that the type of cell receiving such MHC may impact antigen-specific T cell activation.

Intercellular transfer of MHC is mediated not only via trogocytosis but also via exosomes, which are nano-sized membrane vesicles released from various cells (12, 15). Because trogocytosis generally occurs rapidly in a cell–cell contact-dependent manner, it has been considered to be a distinct mechanism from exosome release (9, 16), although some aspects of trogocytosis and exosome-mediated transfer are quite similar, as described below.

Trogocytosis- or exosome-mediated intercellular MHC transfer can transiently generate MHC-dressed cells, which modulate T cell activation (Table 1). For example, CD8^+^ cytotoxic T lymphocytes (CTLs) dressed with APC-derived MHCI are lysed by neighboring CTLs (2, 4). CD4^+^ T cells dressed with dendritic cell (DC)-derived MHCII work as suppressor APCs (17, 18). These reports suggest that DC-derived MHC molecules are functional on T cells (Table 1), although some MHC molecules may be occupied by the TCR. Likewise, DCs also acquire MHC molecules from neighboring DCs, and the transferred MHC molecules are functional on the recipient DCs. Because recent findings on T cells dressed with MHC is well summarized by other review papers (11, 14), this review focuses on the DCs or non-professional APCs dressed with MHCI or MHCII, which acquire APC-like function, and also discusses the differences and similarities between trogocytosis and exosome-mediated transfer.

**Table 1:** **Overview of intercellular MHC transfer**.

Donor cell	Recipient cell	Mechanism	Function (Ref)
**INTERCELLULAR MHCI TRANSFER**			
APCs (DCs)	CD8^+^ T cells	Trogocytosis (TCR-mediated)	Target for neighboring CTLs: fratricide (2, 4)
			TCR downregulation (7)
			Unknown (3)
Live tumor cells	CD8^+^ T cells	Trogocytosis (TCR-mediated)	Target for neighboring CTLs: fratricide (19)
			Enhancement of CTL activity? (20)
			Suppression of CTL activity? (21)
			Stripping MHCI off target tumor cells (8)
APCs	CD4^−^CD8^−^Tregs	Trogocytosis (TCR-mediated)	Antigen presentation for CD8^+^ T cell suppression (22)
DCs	CD4^+^ T cells	Trogocytosis (TCR-mediated bystander)	Antigen presentation for CD8^+^ T cell activation (23)
Live tumor cells	NK cells	Trogocytosis (KIR-mediated)	Suppression of neighboring NK cells (24)
			Unknown (25, 26)
Splenocytes	NK cells	Unknown	Enhancement of killer activity (27)
DCs	DCs	Trogocytosis	Antigen presentation for CD8^+^ T cell activation: cross-dressing (28–30)
DCs, ECs	DCs	Exosomes	Antigen presentation for CD8^+^ T cell activation: cross-dressing (31, 32)
Live tumor cells	DCs	Exosomes	Antigen presentation for CD8^+^ T cell activation (33)
Live tumor cells	DCs	Trogocytosis?	Target for neighboring CTLs (34)
Dead tumor cells	DCs, pDCs	Trogocytosis	Antigen presentation for CD8^+^ T cell activation (35–37)
**INTERCELLULAR MHCII TRANSFER**			
mTECs	Thymic DCs	Unknown	Antigen presentation for central tolerance (38–40)
APCs (DCs)	CD4^+^ T cells	Trogocytosis (TCR-mediated)	Sustaining of TCR signaling (41, 42)
			Antigen presentation for CD4^+^ T cell suppression (17, 18)
			Antigen presentation for CD4^+^ T cell activation (43–45)
APCs (DCs)	CD4^+^ T cells	Exosomes	Antigen presentation for CD4^+^ T cell suppression (46, 47)
			Unknown (48)
APCs	CD4^+^ Tregs	Trogocytosis (TCR-mediated)	Antigen presentation for CD4^+^ T cell suppression (44)
DCs	CD8^+^ T cells	Trogocytosis (TCR-mediated bystander)	Antigen presentation for CD4^+^ T cell activation? (3, 49)
DCs	NK cells	Trogocytosis	Antigen presentation for CD4^+^ T cell suppression (50)
DCs	ILC2s	Trogocytosis	Antigen presentation for CD4^+^ T cell activation (51)
DCs	LNSCs	Trogocytosis and exosomes	Antigen presentation for CD4^+^ T cell suppression (52)
DCs	DCs	Exosomes	Antigen presentation for CD4^+^ T cell activation (31, 53, 54)
Dead tumor cells	DCs	Trogocytosis?	Antigen presentation for CD4^+^ T cell activation (55)
APCs	DCs	Unknown	No antigen-presenting activity (56)

*APCs, antigen-presenting cells; CTLs; cytotoxic T lymphocytes; DCs, dendritic cells; ECs, endothelial cells; ILC2s, group 2 innate lymphoid cells; KIR, killer cell immunoglobulin-like receptor; LNSCs, lymph node stromal cells; MHCI, MHC class I; MHCII, MHC class II; mTECs, medullary thymic epithelial cells; NK, natural killer; pDCs, plasmacytoid DCs; TCR, T cell receptor; Tregs, regulatory T cells*.

## Antigen Presentation by MHCI-Dressed Cells: Cross-Dressing

**Figure 1:**
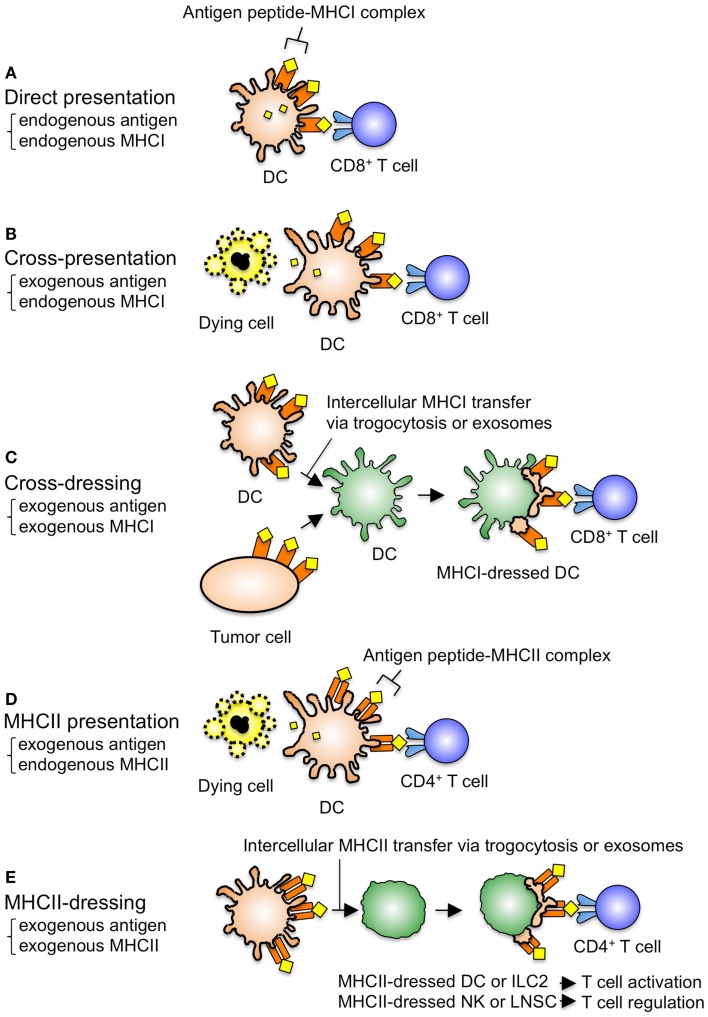
**Canonical and non-canonical antigen presentation pathways**. **(A)** When DCs are virally infected, these DCs present the endogenous viral antigens on the endogenous MHCI molecules to CD8^+^ T cells. **(B)** When certain DC subsets such as mouse CD8α^+^ DCs and human BDCA3^+^ DCs engulf dying cells, these DCs can present the exogenous antigens on the endogenous MHCI molecules to CD8^+^ T cells, which is called cross-presentation. **(C)** When DCs acquire the exogenous antigen–MHCI complexes from neighboring DCs and/or tumor cells, these DCs activate CD8^+^ T cells via the dressed MHCI without requiring processing, which is called cross-dressing. **(D)** When DCs engulf dying cells, these DCs present the exogenous antigens on the endogenous MHCII molecules to CD4^+^ T cells. This is the normal presentation of exogenous antigens. **(E)** When DCs or ILC2s expressing co-stimulatory molecules acquire the exogenous antigen–MHCII complexes from neighboring DCs, these MHCII-dressed cells induce CD4^+^ T cell activation. On the contrary, when NK cells or LNSCs not expressing co-stimulatory molecules acquire the exogenous antigen–MHCII complexes from neighboring DCs, these MHCII-dressed cells suppress CD4^+^ T cell activation. Intercellular MHC transfer is mediated via trogocytosis and/or exosomes. Trogocytosis is defined as an intercellular transfer of plasma membrane fragments that occurs rapidly (within minutes) in a cell–cell contact-dependent manner. Exosomes can be transferred at a distance and diffuse slowly (over several hours).

Dendritic cells have long been known to present MHCI-bound antigens to CD8^+^ T cells through two main pathways: direct presentation of endogenous viral antigens when DCs are virally infected (Figure 1A), and cross-presentation of exogenous antigens from dying tumor cells and virally infected cells that are phagocytosed and processed (57, 58) (Figure 1B). In addition to these canonical antigen presentation pathways, several recent studies proposed a third pathway of antigen presentation in which DCs acquire the preformed peptide–MHCI complexes from neighboring DCs or tumor cells and activate CD8^+^ T cells without any further peptide processing (28, 29, 35, 36, 59, 60) (Figure 1C). This third pathway of antigen presentation was coined “cross-dressing” by Yewdell and Haerfar (61). Although the antigen presentation by DCs dressed with MHCII is also called cross-dressing in some papers (55, 56), this original meaning seems to be the dressed MHCI-mediated antigen presentation (61). Dolan et al. clearly demonstrated the existence of the cross-dressing pathway both *in vitro* and *in vivo* (35). Specifically, it was shown that when H-2^q^ mouse bone marrow-derived DCs (BMDCs) were co-cultured with dying H-2^b^ tumor cell lines expressing ovalbumin (OVA), the H-2^q^ BMDMs acquired the OVA peptide–H-2K^b^ complexes from the tumor cells, and subsequently activated OT-I CD8^+^ T cells expressing the TCR that recognizes the OVA peptide on H-2K^b^ (Figure 1C). Further, by analyzing OT-I proliferation in CD11c-diphtheria toxin receptor (DTA) mice [in which CD11c^+^ DCs are removable by diphtheria toxin (DT) treatment] inoculated with OVA/H-2K^b^ tumor cells, the authors demonstrated that DCs are essential for OT-I proliferation in response to the tumor cell-derived OVA peptide–H-2K^b^ complexes *in vivo* (35). Finally, Wakim and Bevan highlighted the importance of cross-dressing in mouse models of viral infection (29). The authors utilized irradiated (K^d^ × K^b^) F1 mice reconstituted with K^d^ CD11c-DTR bone marrow (BM) cells, in which DCs have only K^d^ and are removable by DT treatment. Following adoptive transfer of OT-I cells into these mice and infection with vesicular stomatitis virus expressing OVA, the authors demonstrated that DCs acquired the OVA peptide–K^b^ complexes from the virally infected cells, and stimulated memory OT-I CD8^+^ T cells, but not naïve OT-I CD8^+^ T cells, *in vivo*. These results suggest that cross-dressing may contribute to effective anti-viral immune responses.

Among mouse DC subsets, CD8α^−^ DCs show higher cross-dressing of neighboring DC-derived MHCI compared with CD8α^+^ DCs (28–30), while CD8α^+^ DCs are essential for the cross-dressing of plasmid DNA vaccine antigens *in vivo* (36). This apparent discrepancy may be ascribed to the difference in type of donor cells (i.e., live DCs, dying tumor cells, etc.) that DCs acquire MHCI from. In addition to these conventional DCs, plasmacytoid DCs (pDCs) are a unique DC subset producing a large amount of type I interferon in response to microbial infection (62), and human pDCs have been also reported to acquire antigen–MHC complexes from tumor cells and to stimulate HLA-A2-restricted T cell proliferation (37).

The frequency of cross-dressing *in vivo* remains to be determined. A number of early reports investigating the cross-presentation pathway (Figure 1B) may have excluded the possibility of the recently emerged cross-dressing pathway (Figure 1C) (57, 58, 63). For example, Kurts et al. engineered an elegant mouse model with which to demonstrate the cross-presentation pathway (64, 65). First, the authors generated the RIP (rat insulin promoter)-mOVA transgenic K^b^ mouse that expresses membrane-bound form of OVA in pancreatic islet β cells and renal proximal tubular cells. RIP-mOVA mice were lethally irradiated and received K^b^ BM cells or K^bm1^ BM cells, where K^bm1^ is a K^b^ mutant that does not present OVA peptide to OT-I cells. After adoptive transfer of OT-I cells into these mice, the authors observed the migration of OT-I cells into renal lymph nodes (LN) of RIP-mOVA mice receiving K^b^ BM cells, but not of the mice receiving K^bm1^ BM cells (64, 65). These results clearly indicate that endogenous MHCI on BM-derived APCs is essential for exogenous antigen presentation. If cross-dressing occurred in this model, the authors would have observed OT-I cell migration in the RIP-mOVA mice receiving K^bm1^ BM cells.

On the other hand, several early studies showed that cross-presentation was not required for priming of CD8^+^ T cells against some exogenous antigens (33, 66, 67). For example, Kundig et al. reported that tumor cells directly induce CTLs *in vivo*, and host MHCI is not involved in this process (66). Wolfers et al. also observed that immunogenic tumor cells directly prime tumor-specific T cells in TAP (transporter associated with antigen presentation)-deficient mice (33). However, these studies do not exclude the possibility that host DCs acquire MHCI from tumor cells for the antigen presentation.

Taken together, it is possible that cross-dressing may occur *in vivo* only under pathological conditions such as during viral infection and cancer. Further, the phenomenon of cross-dressing may explain exogenous antigen presentation to CD8^+^ T cells in mouse models where cross-presentation does not occur.

It is also intriguing to address whether intercellular MHCI transfer impacts donor cell function. As described below, only a small percent of MHCI on donor cells can be transferred to recipient cells (2, 7). Thus, the donor cells seem to retain a sufficient amount of MHCI on their cell surface even after the transfer. However, interestingly, Chung et al. recently reported that low-avidity CTLs strip MHCI off target tumor cells via the mechanism of trogocytosis without killing, resulting in an interference with high-avidity CTLs in tumor lysis (8). It remains unknown whether donor DCs lose the antigen-presenting activity after the release of their MHC molecules to recipient DCs.

## Antigen Presentation by MHCII-Dressed Cells

MHCII is restrictedly expressed on professional APCs where it presents exogenous antigens to CD4^+^ T cells (Figure 1D) (68). In the thymus, intercellular MHCII transfer was observed between medullary thymic epithelial cells (mTECs) and DCs (38, 39). This process is proposed to increase the probability of autoreactive T cells encountering rare antigens for tolerance induction (40, 69). In the periphery, during the interaction between APCs and CD4^+^ T cells, the TCR on the latter trogocytoses MHCII. Because T cells do not express co-stimulatory molecules, MHCII-dressed CD4^+^ T cells induce tolerance in neighboring CD4^+^ T cells, terminating these T cell responses (17, 18). On the contrary, several reports show that CD4^+^ T cells trogocytose not only MHCII but also CD80, and these CD4^+^ T cells dressed with MHCII and CD80 work as APCs for the amplification of CD4^+^ T cell proliferation (43–45). Together, existence of co-stimulatory molecules on MHCII-dressed cells determines these cell functions. Several recent studies reported that intercellular MHCII transfer occurs not only between DCs and T cells, but also between various cells such as DCs and DCs (31, 53, 54, 56), DCs and NK cells (50), DCs and lymph node stromal cells (LNSCs) (52), and DCs and group 2 innate lymphoid cells (ILC2s) (51) (Table 1).

The expression and function of MHCII on mouse NK cells have not been fully understood. We previously showed that mouse conventional NK cells do not synthesize MHCII, rather these cells rapidly acquire MHCII from co-cultured DCs (50). Moreover, NK cells dressed with the OVA peptide–MHCII complexes neither express nor acquire co-stimulatory molecules such as CD80 and CD86, and therefore, suppress but do not induce OT-II CD4^+^ T cell proliferation, suggesting a negative regulation of acquired immunity by NK cells (Figure 1E) (50). NK cells also acquire MHCII from co-cultured B cells; however, the level of MHCII on these NK cells was lower than that on NK cells co-cultured with DCs, suggesting that NK cells preferentially acquire MHCII from DCs (50).

LNSCs have been considered to only play an architectural role in LN construction and homeostasis; however, it is now known that these cells play an important role in peripheral T cell tolerance (70). Dubrot et al. recently showed that LNSCs *per se* synthesize MHCII, but also acquire MHCII from DCs *in vitro* and *in vivo* (52). Like MHCII-dressed NK cells (50), MHCII-dressed LNSCs neither express nor acquire co-stimulatory molecules, resulting in promotion of CD4^+^ T cell apoptosis rather than proliferation. These data suggest that LNSCs maintain peripheral CD4^+^ T cell tolerance via DC-derived peptide–MHCII complexes in LNs (Figure 1E).

ILC2s are recently discovered innate lymphoid cells that produce IL-5, IL-9, and IL-13 and support type-2 immune responses such as allergy and anti-parasite immunity (71). Nevertheless, antigen presentation by ILC2 is not fully understood. Very recently, Oliphant et al. showed that ILC2s express MHCII and also acquire MHCII through trogocytosis *in vivo* (51). ILC2s also express CD80 and CD86 and polarize CD4^+^ T cells toward a Th2 phenotype by antigen presentation in conjunction with type-2 cytokine production. Thus, the dressed MHCII may contribute to type-2 immune responses *in vivo* (Figure 1E).

Given that intercellular transfer of MHCII is observed between DCs and various immune cells, it would be intriguing to address the diffusion of APC-derived MHCII *in vivo*. In fact, such experiments may uncover a novel cell subset capable of activating or regulating CD4^+^ T cells via the preformed antigen peptide–MHCII complexes.

## Mechanism of MHC Trogocytosis

### Do DC receptors mediate MHC trogocytosis?

The molecular mechanism underlying the trogocytosis of MHC by T cells has been well studied (3, 6, 7); however, the mechanism controlling this process in DCs remains to be identified. Of note, Martinez-Martin et al. have reported that TCRs trogocytose MHCI from APCs via small GTPases such as TC21 and RhoG previously known to be associated with phagocytosis. This finding led to the hypothesis that trogotytosis may actually represent immature phagocytosis (7). Therefore, DCs may trogocytose MHC by using phagocytic receptors. Harshyne et al. previously reported that plasma membrane transfer between live monkey DCs was inhibited *in vitro* by a polyanionic reagent, suggesting that scavenger receptors may contribute to the membrane transfer (60), although this has not been confirmed. Li et al. showed that mouse splenic CD8α^+^ DCs are essential for cross-dressing of plasmid DNA vaccine antigen *in vivo* (36). Of note, CD8α^+^ DCs are a unique DC subset that efficiently phagocytose apoptotic cells and perform cross-presentation (57, 58). In addition, these DCs have been reported to recognize dying cells via CLEC9A/DNGR-1 (72, 73), Treml4 (74), and Tim-3 (75); therefore, these receptors may acquire MHCI from dying cells. In contrast, Smyth et al., and Wakim and Bevan independently reported that splenic CD8α^−^ DCs, rather than CD8α^+^ DCs, showed more efficient cross-dressing of neighboring DC-derived MHCI (28–30). In these studies it remains unknown whether the cross-dressing activity is ascribed to the ability to acquire MHCI. Thus, although T cells solely use the TCR to acquire MHC (4, 5, 14), DCs may use different receptors to acquire MHCI from various donor cells.

Additionally, it is unknown if any membrane protein other than MHC is acquired by DCs, although it is unlikely that DCs specifically recognize and acquire only MHCI/II. More likely, DCs nibble a whole membrane fragment at the site of cell–cell contact similar to a model that has been proposed for T cell trogocytosis (4, 16). Indeed, the TCR on CD8^+^ T cells binds MHCI on DCs, and nibbles the MHCI-containing membrane fragments, in which MHCII is also contained (3, 49). Likewise, CD4^+^ T cells trogocytose not only MHCII but also MHCI from DCs through the bystander mechanism (23) (Table 1). Thus, any membrane proteins at the cell–cell contact area would be transferred to DCs. The reason only MHC molecules may appear to be transferred is probably because MHC molecules are highly expressed on donor cells and are easily detected. Only a small percent (at most 10%) of highly expressed cell surface molecules on donor cells can be transferred to recipient cells, as shown by the fluorescence intensity of flow cytometry and confocal microscopy data in many reports (2, 7, 29, 35, 36, 50, 76). Therefore, it is difficult to detect the trogocytosis of molecules with low expression on donor cells, even if trogocytosis occurs. Identification of the DC receptor(s) and ligand(s) required for the trogocytosis of MHCI will resolve these issues.

### Is trogocytosis different from exosome-mediated transfer?

Exosomes are nano-sized membrane vesicles released by a wide variety of cell types (15). Many reports have shown that MHCI-bearing exosomes are secreted from APCs and several tumor cell lines, and that these exosomes play important roles in anti-tumor immunity (15, 77). For example, tumor-derived exosomes can activate CD8^+^ T cells when co-cultured with DCs (33, 78). In these experiments, the exosomal MHCI is not critical, suggesting that tumor antigen alone, but not tumor-derived MHCI, is required for DC-mediated antigen presentation (33, 78). Meanwhile, DC-derived exosomes bear MHCI and co-stimulatory molecules, and *per se* are able to induce CD8^+^ T cell proliferation (15).

Wakim and Bevan conducted experiments to carefully address the possibility that cross-dressing is mediated via MHCI-bearing exosomes (29). In these experiments, it was observed that DC-derived exosomes alone, but not those co-cultured with H-2K^bm1^ DCs, induce OT-I CD8^+^ T cell proliferation, leading to the speculation that exosomes may be internalized by and degraded within DCs. Furthermore, the authors showed that DCs co-cultured at a distance (using a transwell system) lost cross-dressing activity, suggesting that the intercellular transfer of the peptide–MHCI complexes between DCs is mediated via cell–cell contact-dependent trogocytosis rather than exosomes (29).

MHCII-bearing exosomes are also secreted from DCs and possess functional capacity (15, 79). For instance, Théry et al. reported that DC-derived MHCII-bearing exosomes acquired by MHCII-deficient DCs can stimulate antigen-specific CD4^+^ T cell proliferation *in vitro* (54). Dubrot et al. observed that MHCII transfer from DCs to LNSCs is cell–cell contact-dependent, suggesting trogocytosis. Nevertheless, the authors do not exclude the possibility that DC-derived exosomes may also contribute to this process because LNSCs co-cultured with DC-derived exosomes also became MHCII-positive and activated CD4^+^ T cells (52).

As described above, trogocytosis was originally defined as an intercellular transfer of plasma membrane that occurs rapidly (within minutes) in a cell–cell contact-dependent manner. In contrast, exosomes can be transferred at a distance and diffuse slowly (over several hours) (10, 12). Indeed, the TCR and NK receptors acquire MHC and related molecules from donor cells within minutes in a cell–cell contact-dependent manner, and this does not occur when donor cells and recipient cells are co-cultured at a distance in a transwell plate (2, 7, 50, 76, 80), which is typical of trogocytosis. However, acquisition of MHC by DCs takes several hours (28, 29, 31, 35, 36). To make matters more complicated, Mittelbrunn et al. recently showed that T cells secrete exosomes when a functional immunological synapse is formed at the APC contact site, and that this release of exosomes is abrogated by a transwell culture system (81). Together, these reports make it difficult to discriminate between trogocytosis and exosome-mediated transfer. As there are a variety of types of exosomes and exosome-like microvesicles (15), it is reasonable to hypothesize that trogocytosis may actually be mediated via several unique mechanisms, and furthermore, that some instances of exosome transfer and trogocytosis may be mediated via a shared mechanism.

## Conclusion

There is no doubt that DCs play a crucial role in antigen processing and presentation. However, it was unforeseen that the antigen peptide–MHC complexes on DCs can be transferred to neighboring DCs or non-APCs where the preformed peptide–MHC complexes are involved in antigen-specific T cell activation without requirement for further processing. These MHC-dressed cells expressing or not expressing co-stimulatory molecules could contribute to T cell activation or suppression, respectively, which may play an important role in fine-tuning signals from DCs *in vivo*. As the occurrence of this transfer has been well established, the leading question remaining is how exactly the MHC molecules are transferred. It also remains unknown whether donor cell-derived membrane fragments including MHC can be fused or are merely attached to the membrane of recipient cells. Identification of the molecular mechanisms underlying MHC trogocytosis and exosome transfer will enable us to perturb these pathways and further address the physiological relevance of non-canonical antigen presentation *in vivo*.

## Conflict of Interest Statement

The author declares that the research was conducted in the absence of any commercial or financial relationships that could be construed as a potential conflict of interest.
